# The Significance of Circular RNA DDX17 in Prostate Cancer

**DOI:** 10.1155/2020/1878431

**Published:** 2020-08-20

**Authors:** Qi Lin, Jian Cai, Qin-Quan Wang

**Affiliations:** ^1^Department of Urology, The First Affiliated Hospital of Wenzhou Medical University, Wenzhou, China; ^2^Department of Andriatry, The First Affiliated Hospital of Wenzhou Medical University, Wenzhou, China

## Abstract

Circular RNA DDX17 (circDDX17) has been demonstrated as a tumor suppressor in colorectal cancer. However, mechanisms underlying circDDX17 effects in cases of prostate cancer (PCa) are not well understood. Thus, herein, we determined measures of circDDX17 expression by use of the TCGA database. Expression of circDDX17 in prostate cancer-afflicted tissue samples was determined by qRT-PCR. Functionally, circDDX17 induced remarkable inhibition of cell colonizing ability, invasion, and epithelial-mesenchymal transition (EMT) progression in vitro. Mechanistically, dual-luciferase reporter assays, RNA immunoprecipitation, and RNA pull-down experiments helped verify interactions between circDDX17 and miR-346. Low expression of circDDX17 occurred in TCGA PCa samples. Furthermore, circDDX17 expression was downregulated significantly in PCa. These results suggested that circDDX17 suppressed PC cell mobility, proliferation, and invasion. Mechanistic experiments indicated that circDDX17 might serve as a ceRNA of miR-346 to relieve repressive effects of miR-346 upon phospholysine phosphohistidine inorganic pyrophosphate phosphatase (LHPP). LHPP expression itself was downregulated in TCGA PCa samples. Overall, our findings indicated that the circDDX17/miR-346/LHPP pathway inhibited the progression of prostate cancer and that circDDX17 may be a new potential therapeutic or diagnostic target for treating and diagnosing prostate cancer. As our study also demonstrated for the first time that LHPP might act as an anticancer gene in prostate cancer, the findings could have wide-ranging implications for the treatment of this affliction.

## 1. Introduction

Prostate cancer (PCa) is the most prevalent type of cancer afflicting males and contributes to huge societal burden for many countries [1]. Although recent research has furthered the accuracy of PCa diagnoses and effectiveness of therapies, the five-year survival rate for PCa patients and long-term prognoses are still undesirable or inconvenient [2, 3]. Therefore, there is an urgent need to investigate additional molecular mechanisms underlying PCa such as to help confirm novel diagnostic or therapeutic targets.

Circular RNAs (circRNAs) are a novel class of endogenous noncoding RNAs. They do not have 5′ or 3′ ends and covalently link to form a closed circular structure [4]. This closed loop structure, known as a “back-splicing,” is generated from backsplicing [5]. circRNAs are expressed widely in different cell types and species, and greater than 20,000 circRNAs have been discovered in eukaryotes as facilitated by development and advancement of sequencing technologies and bioinformatics [6]. Exonic circRNAs are the most abundant type of circRNAs and are mainly located in the cytoplasm because they only have exon sequences, whereas intron circRNAs are generally located in nuclei [7]. As of recently, exonic circRNAs are the most thoroughly researched of all types of circRNAs. One type of circRNA, circDDX17, was first reported to act as a tumor suppressor in examinations of samples afflicted by colorectal cancer [8]. However, the exact roles of circDDX17 and its influences upon the dynamics and mechanisms underlying PCa remain unknown.

Phospholysine phosphohistidine inorganic pyrophosphate phosphatase (LHPP) was first identified in the brain tissue of swine [9, 10]. Hindupur et al. reported that LHPP acted as a tumor suppressor and inhibited human hepatocellular carcinoma (HCC) progression [11]. Zheng et al. also demonstrated that silencing of LHPP in cervical cancer promoted subsequent cell metastasis, apoptosis, and proliferation via the regulation of AKT [12]. However, to date, the roles underlying the influence upon the mechanisms and dynamics of LHPP in PCa are yet to be elucidated.

Therefore, we sought to determine expression of circDDX17 between tumor-afflicted tissue samples and corresponding adjacent nontumor-afflicted samples. Furthermore, we also sought to illuminate the role of circDDX17 in metastasis and epithelial–mesenchymal transition of PCa cell lines. Additionally, we sought to use bioinformatics and fluorescence in reporting experiments with the expectation that we would reveal an interaction between circDDX17, miR-346, and LHPP. We hypothesized that circDDX17 would play an oncogenic role in PCa by way of competing with miR-346 such as to enhance LHPP expression and then subsequently repress PCa progression. In conclusion, we sought to examine if and expected that circDDX17 could be a potential diagnostic or therapeutic target for PCa patients.

## 2. Methods and Materials

### 2.1. Bioinformatics Analyses

The expression profiles of DDX17 in PCa-afflicted samples were obtained from TCGA dataset (http://gepia.cancer-pku.cn/index.html). UCSC (http://genome.ucsc.edu/; [13] was used to search for the gene for LHPP. Circinteractome (https://circinteractome.nia.nih.gov) and TargetScan (http://www.targetscan.org/vert_72/) results were applied to facilitate the prediction of miRNAs that had complementary base pairing sites with circDDX17 and LHPP.

### 2.2. Clinical Tissue Specimens

Twenty pairs of PCa-afflicted tissues and corresponding adjacent noncancerous tissues were harvested. All patients provided signed, written informed consent. The patients all had undergone surgery in the First Affiliated Hospital of Wenzhou Medical Hospital (Wenzhou, China). Two pathologists independently histopathologically evaluated and characterized all individual tissue specimens. Freshly resected tissue specimens were snap-frozen in liquid nitrogen and stored at -80°C until further use. All uses of clinical tissue specimens were subsequently performed according to the Declaration of Helsinki and were approved by The Institutional Review Board of the First Affiliated Hospital of Wenzhou Medical Hospital.

### 2.3. Cell Lines and Culture

22Rv1 and PC-3 cell lines were obtained from the American Type Culture Collection (Manassas, VA). 22Rv1 cells were cultured in RPMI-1640 Medium (Invitrogen) supplemented with 10% fetal bovine serum (FBS, Invitrogen). PC-3 cells were cultured in F-12K Medium (Invitrogen) supplemented with 10% FBS (Invitrogen). We cultured all of these cells in a humidified atmosphere at 37°C with 5% CO_2_.

### 2.4. Total RNA Extraction and qRT-PCR

Total RNA was isolated from prostate tissue samples and cell lines with TRIzol (TaKaRa, China) following all manufacturer protocols. We treated total RNA with DNase I (TaKaRa, Dalian, China) to remove genomic DNA. Reverse transcription of purified RNA was carried out using random primer sets and standardized reaction conditions with the use of PrimeScript RT Master Mix (Applied Biosystems, Foster City, CA). Quantitative real-time PCR (qRT-PCR) was carried out on the ABI7300 system (Applied Biosystems) following all manufacturer protocols. Glyceraldehyde 3-phosphate dehydrogenase (GAPDH) or U6 was used as internal controls. The 2^-*ΔΔ*Ct^ method was used to assess relative transcription alterations according to a previous study [14]. qPCR primer sequences are shown in Table 1.

### 2.5. Transfection of RNAi and Plasmids

Two LHPP-siRNAs, a negative control (NC) for the LHPP-siRNAs, plasmids pPG-miR-Blasticidin with hsa-miR-346 mimics (pPG-miR-346) or hsa-miR-346 inhibitor (pPG-anti-miR-346) or their NC (pPG-miR-NC), were all designed, synthesized, and supplied by GenePharma, China. Sequences of siRNAs were as follows: 5′-TTCTCCGAACGTGTCACGT-3′ for si-NC, 5′-UUCUCCGAACGUGUCACGUTT-3′ for si-NC, 5′-CCACAAATTTGGAGCAAGA-3′ for si-circDDX17-1, and 5′-GAAAAAGACCACAAATTTG-3′ for si-circDDX17-2 [8]; 5′-CAACCCAAACUGUGUGGUA-3′ for si-LHPP-1, and 5′-CAUGAAGGCGCUUGAGUAU-3′ for si-LHPP-2 [11]. The pcDNA3.1-circDDX17 and empty vector was designed, synthesized, and supplied from Sangon Biotech, China. Cells were place upon six-well plates until confluence and then were transfected with siRNAs or plasmids by using Lipofectamine 3000 (Invitrogen, USA) following manufacturer protocols. After 48 h, cells were obtained for follow-up experiments.

### 2.6. Transwell Invasion Assays

Cell invasion was determined by using transwell chambers (Corning, New York, NY) following all manufacturer protocols. Cells on upper surfaces of the transwell chambers were removed using cotton swabs after 24 h of incubation. Thereafter, cells below the membrane were fixed using methanol for 10 m and stained by crystal violet. Five randomly selected fields were selected for cell counting.

### 2.7. Cell Counting Kit-8 Assay

After transfection of pcDNA-NC or pcDNA-circDDX17 for 48 h, 22Rv1 and PC-3 cell lines were seeded into 96-well plates at 5 × 10^3^ cells/well and were incubated over prolonged time periods (0, 24, 48, 72, and 96 h) prior to the addition of 10 *μ*L of Cell Counting Kit-8 (CCK8) solution per well for 1 h. Absorbance (450 nm) was assessed using a Synergy microplate reader (BioTek, Winooski, VT).

### 2.8. Luciferase Reporter Assay

22Rv1 and PC-3 cells were cotransfected with pmiR-GLO-NC, pmiR-GLO-circDDX17-wt or pmiR-GLO-circDDX17-mut, pmiR-GLO-LHPP-wt or pmiR-GLO-LHPP-wt or pmiR-GLO-LHPP-mut (Sangon, Biotech), and an internal control pRL-TK (Promega). Thereafter, pPG-miR-346 or pPG-miR-NC were cotransfected into 22Rv1 and PC-3 cells. Luciferase activities were evaluated by using a dual-luciferase reporter assay kit (Promega, USA) following all manufacturer protocols. Renilla luciferase activity acted as the means to assess levels for normalization.

### 2.9. RNA-Binding Protein Immunoprecipitation Assay

RNA-binding protein immunoprecipitation assays (RIP) were carried out by using the Magna RIP RNA-Binding Protein Immunoprecipitation Kit (Millipore, Billerica, MA, USA). The Ago2 plasmid or vector was transfected into 22Rv1 and PC-3 cells. Next, cells were pelleted and resuspended in 100 *μ*L of RIP lysis buffer containing with it a protease inhibitor cocktail and RNase inhibitors. Five *μ*g of antibody against Ago (Millipore) or rabbit IgG-coated beads were added into cell lysates (200 *μ*L) and then rotated overnight while held at a constant temperature of 4°C. Immunoprecipitated RNA was extracted using the RNeasy MinElute Cleanup Kit (Qiagen) posttreatment of lysates with proteinase K buffer. Reverse transcription was performed by using PrimeScript RT Master Mix (TaKaRa). qRT-PCR was performed to facilitate detection of circDDX17 abundances.

### 2.10. Biotin-Labelled miRNA Pull-Down Assay

A pull-down assay was carried out as previously described [15]. Briefly, 1 × 10^7^ 22Rv1 and PC-3 cells were obtained, lysed, and sonicated. Cell lysates were incubated with the circDDX17 probe or oligoprobe at 4°C, overnight. The circDDX17 probes were crosslinked by coincubating the circDDX17 probe with C-1 magnetic beads (Life Technologies) at 25°C for 2 h. RNA complexes bound to beads were then eluted and extracted using the RNeasy Mini Kit (Qiagen) for qRT-PCR after washing with wash buffer. The biotinylated circDDX17 probe was purchased from Sangon (Shanghai, China).

### 2.11. Immunofluorescence Analysis

Cells were seeded upon glass coverslips in six-well plates and were then fixed with 4% paraformaldehyde for 20 min. Then, 0.1% Triton X-100 was used to permeabilize cells for 15 min and blocking was achieved using 5% goat serum in PBS for 1 h. Cells were then incubated in primary antibodies at 4% overnight. Primary antibodies used included E-cadherin and Vimentin (1: 50, Proteintech, USA). Cells were washed with PBST and then incubated in secondary antibody (Abcam, USA) for 1.5 h at 37°C. Levels of immunofluorescence were assessed through photography facilitated by using fluorescence microscopy (Olympus BX51).

### 2.12. Western Blotting

Total proteins were extracted from cells using RIPA Lysis Buffer (Beyotime, China) containing a protease inhibitor cocktail (Beyotime, China) while samples were kept on ice. Total protein from each sample was separated by using SDS-PAGE gels and then were electrotransfered onto 0.45 *μ*m polyvinylidene difluoride (PVDF) membranes (Millipore, USA). Next, membranes were blocked by using 5% skim milk powder in TBST for 2 h at room temperature and were then incubated with primary antibodies at 4% overnight. Finally, membranes were thrice washed with TBST and were then treated with an HRP-conjugated secondary antibody (Beyotime, China). Each of the specific primary antibodies was composed as follows: anti-E-cadherin (1 : 1000, Proteintech, USA), anti-Vimentin (1 : 1000, Proteintech, USA), anti-GAPDH (1 : 1000, Beyotime, China, USA), and anti-LHPP (1 : 500, Proteintech, China).

### 2.13. Colony Formation Assay

Cells were seeded at densities of 1000 cells·well^−1^ into a six-well plate and cultured for 8 days. Cells were washed with PBS and then fixed with 4% paraformaldehyde for 20 min. Finally, cells were stained with a 0.5% crystal violet solution for 10 min. Colonies were enumerated under microscopy.

### 2.14. Statistical Analyses

All data are presented as mean ± standard deviation (±SD). Statistical analyses were calculated using GraphPad Prism 5. Levels of expression of circDDX17 and miR-346 in tumor-afflicted tissue samples and adjacent nontumorous tissue samples were analyzed by using paired-sample *t*-tests. Independent-sample *t*-tests were used to analyze differences between groups. All *p* values were calculated as two-sided where ^∗^*p* < 0.050 was considered the level of statistical significance at which the null hypothesis of no differences between treatment groups would be rejected.

## 3. Results

### 3.1. circDDX17 Was Markedly Downregulated in PCa and Facilitated Invasion and Proliferation of PCa-Afflicted Cells

**Figure 1:**
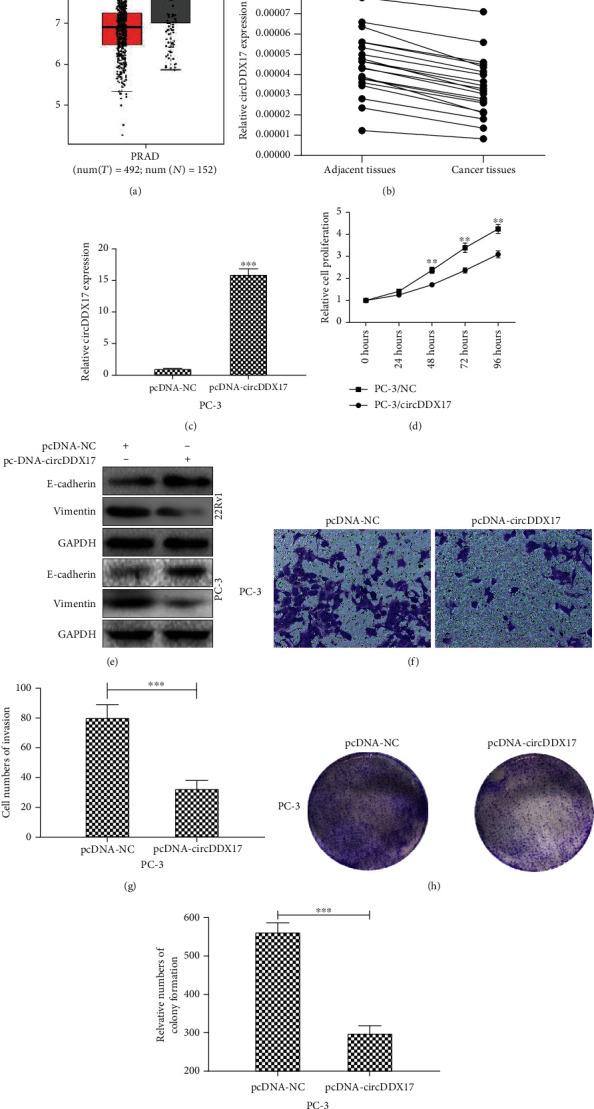
Upregulation of circDDX17 suppresses the migration, EMT, and proliferation of prostatic cancer cells. (a) DDX17 expression in PCa and normal tissues was harvested form TCGA database. Red box represented PCa samples and gray box represented normal tissues. *p* < 0.01. (b) The circDDX17 expression in prostate cancer tissue samples and corresponding adjacent nontumor tissue samples was determined by qPCR. (c) The circDDX17 expression in PC-3 cell lines transfected with pcDNA-NC or pcDNA-circDDX17 was determined by qPCR. (d) Relative cell proliferation in PC-3 cell lines transfected with pcDNA-NC or pcDNA-circDDX17 was detected by CCK8. (e) The protein E-cadherin and Vimentin expression in 22Rv1 and PC-3 cell lines transfected with pcDNA-NC or pcDNA-circDDX17 was measured by Western blotting. (f) The invasion of PC-3 cell lines transfected with pcDNA-NC or pcDNA-circDDX17 was determined by transwell assays. (g) Relative cell numbers of invasion in PC-3 cell lines are shown. (h) The colonizing ability of PC-3 cell lines transfected with pcDNA-NC or pcDNA-circDDX17 was determined by colony formation assays. (i) Relative cell numbers of colony formation in 22Rv1 cell lines are shown. ^∗^*p* < 0.05, ^∗∗^*p* < 0.01, and ^∗∗∗^*p* < 0.001.

According to TCGA database, the expression profile of DDX17 in TCGA PCa samples was determined (Figure 1(a)). The expression profile of DDX17 was reduced in PCa-afflicted samples compared to nonafflicted sampled based upon TCGA database. Furthermore, qPCR was performed to evaluate relative circDDX17 expression in 20 pairs of PCa tissue samples and corresponding adjacent normal samples.  Figure 1(b) demonstrates that relative circDDX17 expression was downregulated in PCa-afflicted tissue samples. To confirm the role of circDDX17 in PCa cells, PC-3 and 22Rv1 cell lines were transfected with plasmids containing pcDNA-circDDX17 or pcDNA-NC. qPCR results demonstrated that expression of circDDX17 increased in 22Rv1 and PC-3 cell lines (Figure 1(c) and Figure S1A). CCK8 assays were used to assess the proliferation of 22Rv1 and PC-3 after transfection of pcDNA-NC or pcDNA-circDDX17, and results indicated that proliferation of 22Rv1 and PC-3 cell lines was repressed (Figure 1(d) and Figure S1B. To investigate the role of circDDX17 in EMT progression, the expression of the EMT marker E-cadherin and Vimentin was determined by Western blotting. As exemplified in Figure 1(e), protein expression of E-cadherin was markedly upregulated and Vimentin expression was markedly reduced in 22Rv1 and PC-3 cell lines transfected with pcDNA-circDDX17. Next, we completed transwell assays to evaluate the degree of invasion of 22Rv1 and PC-3 cells transfected with pcDNA-NC or with pcDNA-circDDX17. Results indicated that measures of invasion of 22Rv1 and PC-3 were both significantly reduced (Figure 1(f) and Figure S1C). Quantitative analyses indicated that differences between pcDNA-NC and pcDNA-circDDX17 were significant (Figure 1(g) and Figure S1D). Finally, we performed colony formation assays to evaluate colonizing abilities of PCa cells. Results indicated that the colonizing ability of PCa cells was changed significantly after transfection of pcDNA-circDDX17 (Figures 1(h) and 1(i) and Figures S1E and F). These data revealed that increased circDDX17 expression might have repressed the subsequent progression of PCa.

### 3.2. Reciprocal Repression of circDDX17 and miR-346 in PCa Cell

circRNAs competitively combined with respective complementary miRNA sequences whereby mediation of expression of protein was accomplished by way of having acted as a ceRNA type of mechanism [16]. Li et al. [7] demonstrated that circRNAs are mainly located in the cytoplasm. Thus, we hypothesized that circDDX17 could have acted as a molecular sponge and thereby regulated miRNAs in the cytoplasm. We found that miR-346 had a putative complementary sequence with circDDX17 by using predictions derived from online database data (Circinteractome, https://circinteractome.nia.nih.gov). Recent research reported that miR-346 promoted the progression of various types of cancers [17–19]. In support of our findings, miR-346 was also reported to have been overexpressed in samples afflicted by PCa [20].

**Figure 2:**
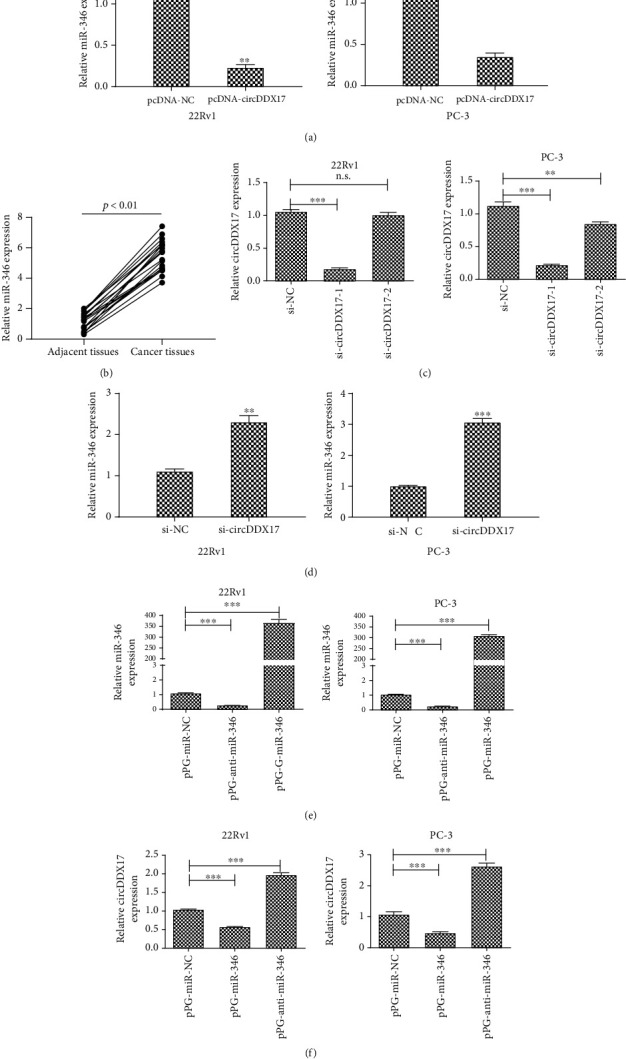
Competitive expression between circDDX17 and miR-346. (a) Relative miR-346 expression in prostate cancer tissue samples and corresponding adjacent nontumor tissue samples was determined by qPCR. (b) The miR-346 expression in 22Rv1 and PC-3 cell lines transfected with pcDNA-NC or pcDNA-circDDX17 was determined by qPCR. (c) Relative circDDX17 expression in 22Rv1 and PC-3 cell lines transfected with si-NC, si-circDDX17-1 or si-circDDX17-2 was measured by qPCR. si-circDDX17-1 was used in follow-up experiments. (d) Relative miR-346 expression in 22Rv1 and PC-3 cell lines transfected with si-NC or si-circDDX17 was detected by qPCR. (e) Relative miR-346 expression in 22Rv1 and PC-3 cell lines transfected with pPG-miR-NC, pPG-anti-miR-346, or pPG-miR-346 was detected by qPCR. (f) Relative circDDX17 expression in 22Rv1 and PC-3 cell lines transfected with pPG-miR-NC, pPG-anti-miR-346, or pPG-miR-346 was detected by qPCR. ^∗^*p* < 0.05, ^∗∗^*p* < 0.01, and ^∗∗∗^*p* < 0.001.

To investigate relationships between circDDX17 and miR-346, we used qPCR to assess measures of expression of miR-346 in 22Rv1 and PC-3 cell lines posttransfection with pcDNA-NC or with pcNDA-circDDX17. As seen in Figure 2(a), the expression of miR-346 was obviously reduced in 22Rv1 and PC-3 cell lines transfected with pcDNA-circDDX17 compared to samples transfected with pcDNA-NC. qPCR was used to determine the expression of miR-346 expression in prostate-afflicted cancer tissue samples. qPCR analyses indicated that relative miR-346 expression was significantly upregulated in PCa-afflicted tissue samples compared to unafflicted tissues. Next, we established whether or not si-NC, si-circDDX17-1, and si-circDDX17-2 could be used to silence the expression of circDDX17. In Figure 2(c), it can be seen that circDDX17 expression was markedly changed by the application of si-circDDX17-1. Si-cricDDX17-1 was thus used in follow-up experiments. The subsequent silencing of circDDX17 was found to have promoted miR-346 expression in both 22Rv1 and PC-3 cell lines (Figure 2(d)). To explore the function of miR-346, we constructed pPG-miR-NC, pPG-anti-miR-346, and pPG-miR-346 to facilitate silencing of miR-346 in 22Rv1 and overexpression of miR-346 in PC-3 cell lines. qPCR confirmed the effect of pPG-miR-346 and pPG-anti-miR-346 (Figure 2(e)). Notably, expression of circDDX17 was obviously silenced by pPG-miR-346 and was overexpressed by pPG-anti-miR-346 (Figure 2(f)). These results confirmed that there was reciprocal repression of circDDX17 and miR-346 in PCa-afflicted cells.

### 3.3. circDDX17 Binds to miR-346 Directly

**Figure 3:**
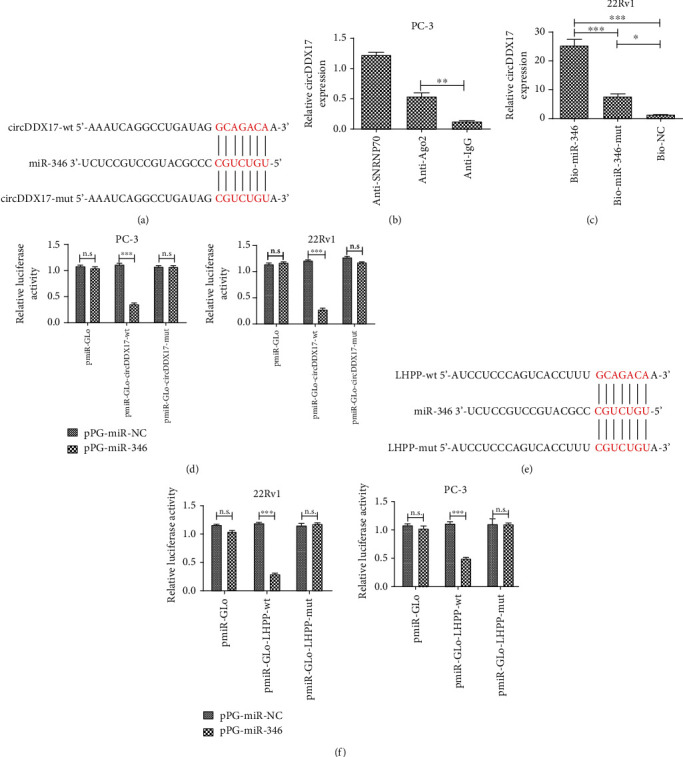
Identification of potential matching sequence in miR-346 and LHPP mRNA. (a) Alignment of miR-346 sequence with circDDX17 and circDDX17 mutated at the potential binding site. (b) Relative luciferase activity in 22Rv1 and PC-3 cell lines cotransfected with pPG-miR-138 (or the empty vector as a control) and the luciferase empty vector (pmiR-GLo) or the vector containing the wild-type circDDX17 (pmiR-GLo-DDX17-wt) or mutant transcripts (pmiR-GLo-DDX17-mut) was detected. All data are shown as the relative ratio of firefly luciferase activity to Renilla luciferase activity. (c) PC-3 cell lines were transfected with biotinylated NC (Bio-NC), biotinylated wild-type miR-346 (BiomiR-346), or biotinylated mutant miR-346 (Bio-miR-346-mut), and biotin-based miRNA pull-down assays were performed after 48 h of transfection. circDDX17 levels were determined by qPCR. (d) Amount of circDDX17 bound to SNRNP70 (a positive control), Ago2, or IgG (a negative control) was detected by qPCR after RIP in 22Rv1 cells. (e) Alignment of LHPP mRNA sequence with miR-346 and miR-346 mutated at the potential binding site. (f) Relative luciferase activity in 22Rv1 and PC-3 cell lines cotransfected with pPG-miR-346 (or the empty vector as a control) and the luciferase empty vector (pmiR-GLo) or the vector containing the wild-type LHPP (pmiR-GLo-LHPP-wt) or mutant transcripts (pmiR-GLo-LHPP-mut) was detected. All data are shown as the relative ratio of firefly luciferase activity to Renilla luciferase activity. ^∗^*p* < 0.05, ^∗∗^*p* < 0.01, and ^∗∗∗^*p* < 0.001.

Putative binding sequences between circDDX17 and miR-346 are shown in Figure 3(a). Through binding to Ago2, a core component of the RNA-induced silencing complex (RISC), miRNAs utilize their gene-silencing function [21]. RIP assays with Ago2 antibody were used to isolate RNA from RISC, and results indicated that circDDX17 was markedly enriched in Ago-2-containing beads in PC-3 cell lines (Figure 3(b)). In addition, miRNA pull-down assays were performed by transfection of biotinylated miR-346, biotinylated miR-346-mut, or biotinylated NC into 22Rv1 cell lines. The results of pull-down assays demonstrated that circDDX17 was capable of being pulled down by miR-346 (Figure 3(c)).

To confirm whether the putative binding site was efficient, a dual-luciferase reporter assay in 22Rv1 and PC-3 cell lines was performed. The luciferase activity of 22Rv1 and PC-3 cell lines decreased after cotransfection with pPG-miR-346+pmiR-Glo-circDDX17-wt but did not decrease with pPG-miR-346+pmiR-Glo or pPG-miR-346+pmiR-Glo-circDDX17-mut (Figure 3(d)). These results suggested that the putative binding site was necessary for reciprocal repression of circDDX17 and miR-346.

### 3.4. circDDX17 Positively Regulates LHPP, a Target of miR-346

**Figure 4:**
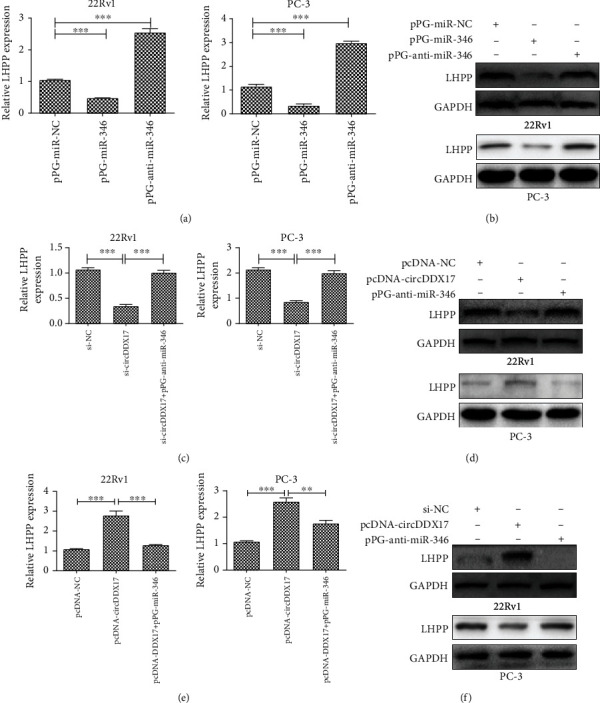
circDDX17 regulates the expression of LHPP through sponging miR-346. (a) Relative LHPP mRNA expression in 22Rv1 and PC-3 cell lines transfected with pPG-miR-NC, pPG-anti-miR-346, or pPG-miR-346 was detected by qPCR. (b) Relative LHPP protein expression in 22Rv1 and PC-3 cell lines transfected with pPG-miR-NC, pPG-anti-miR-346, or pPG-miR-346 was detected by Western blotting. (c) Relative LHPP mRNA expression in 22Rv1 and PC-3 cell lines transfected with si-NC, si-circDDX17, or si-circDDX17+pPG-anti-miR-346 was determined by qPCR. (d) Relative LHPP protein expression in 22Rv1 and PC-3 cell lines transfected with si-NC, si-circDDX17, or si-circDDX17+pPG-anti-miR-346 was determined by Western blotting. (e) Relative LHPP mRNA expression in 22Rv1 and PC-3 cell lines transfected with pcDNA-NC, pcNDA-circDDX17, or pcNDA-circDDX17+pPG-miR-346 was determined by qPCR. (f) Relative LHPP protein expression in 22Rv1 and PC-3 cell lines transfected with pcDNA-NC, pcNDA-circDDX17, or pcNDA-circDDX17+pPG-miR-346 was determined by Western blotting. ^∗^*p* < 0.05, ^∗∗^*p* < 0.01, and ^∗∗∗^*p* < 0.001.

To predict the target protein of miR-346, we searched an online database (TargetScan, http://www.targetscan.org/vert_72/) and used the results to help predict the putative targets of miR-346. As seen in Figure 3(e), the putative binding site of LHPP with circDDX17 was matched in miR-346 sequence. To further confirm if there were interactions between circDDX17, miR-346, and LHPP, follow-up experiments were performed. Firstly, we performed a dual-luciferase reporter assay for 22Rv1 and PC-3 cell lines to confirm that the potential site was functional. Results indicated that luciferase activity was reduced dramatically in 22Rv1 and PC-3 cell lines transfected with pmiR-Glo-LHPP-wt, whereas comparatively, the luciferase activity did not change in cells transfected with pmiR-Glo-LHPP-mut (Figure 3(f)). These data suggested that miR-346 directly combined with the 3′UTR of LHPP in 22Rv1 and PC-3 cell lines. Secondly, the results from qPCR and Western blotting indicated that the levels of mRNA and expression of LHPP proteins were increased in 22Rv1 and PC-3 cell lines transfected with pPG-anti-miR-346, whereas they were decreased in cells transfected with pPG-miR-346 (Figures 4(a) and 4(b)). Thirdly, we investigated whether or not circDDX17 could control LHPP expression through competing with miR-346 in PCa-afflicted cells. Silencing of circDDX17 in 22Rv1 and PC-3 cell lines markedly repressed LHPP. However, pPG-anti-miR-346 could reverse these effects (Figures 4(c) and 4(d)). Upregulation of circDDX17 promoted the levels of mRNA and expression of proteins of LHPP in 22Rv1 and PC-3 cell lines, whereas pPG-miR-346 could induce a reversal of these effects (Figures 4(e) and 4(f)). These results indicated that circDDX17 promoted LHPP expression by way of binding miR-346.

### 3.5. circDDX17 Inhibited Metastasis and Epithelial-Mesenchymal Transition of PCa-Afflicted Cells

**Figure 5:**
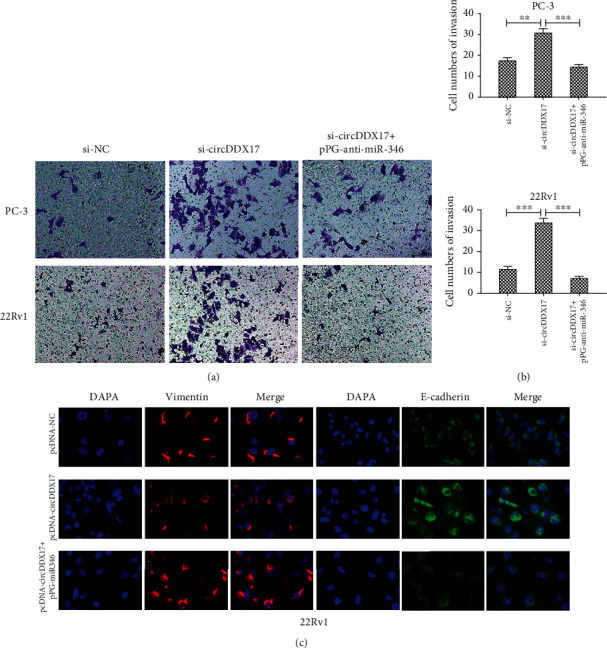
Silencing of miR-346 can suppress the EMT and invasion of prostate cancer cells induced by circDDX17 knockdown. (a) The invasion of PC-3 and 22Rv1 cell lines transfected with si-NC, si-circDDX17, and si-circDDX17+pPG-anti-miR-346 was determined by transwell assays. (b) Relative numbers of invasion were shown. (c) The E-cadherin and Vimentin protein expression in 22Rv1 cell lines transfected with si-NC, si-circDDX17, and si-circDDX17 + pPG-anti-miR-346 was determined by immunofluorescence. ^∗^*p* < 0.05, ^∗∗^*p* < 0.01, and ^∗∗∗^*p* < 0.001.

To investigate the function of circDDX17 in PCa cell metastasis, we performed transwell invasion assays. These assays facilitated the evaluation of effects of circDDX17 knockdown upon the ability of cells to invade. Silencing of circDDX17 in 22Rv1 and PC-3 cells dramatically promoted cell invasion, whereas pPG-anti-miR-346 significantly reduced cell invasion (Figures 5(a) and 5(b)).

To verify whether circDDX17 regulated PCa cell metastasis by inducing EMT, we used immunofluorescence for epithelial marker E-cadherin and mesenchymal marker Vimentin. Results from immunofluorescence assays indicated that overexpression of circDDX17 enhanced E-cadherin expression and weakened the expression of Vimentin. However, pPG-miR-346 could reverse these effects (Figure 5(c) and Figures S1G and H). These data suggested that circDDX17 could transfer epithelial cells to facilitate their transdifferentiation into mesenchymal cells.

### 3.6. miR-346 Improved PCa Cell Metastasis and Epithelial-Mesenchymal Transition Progression

**Figure 6:**
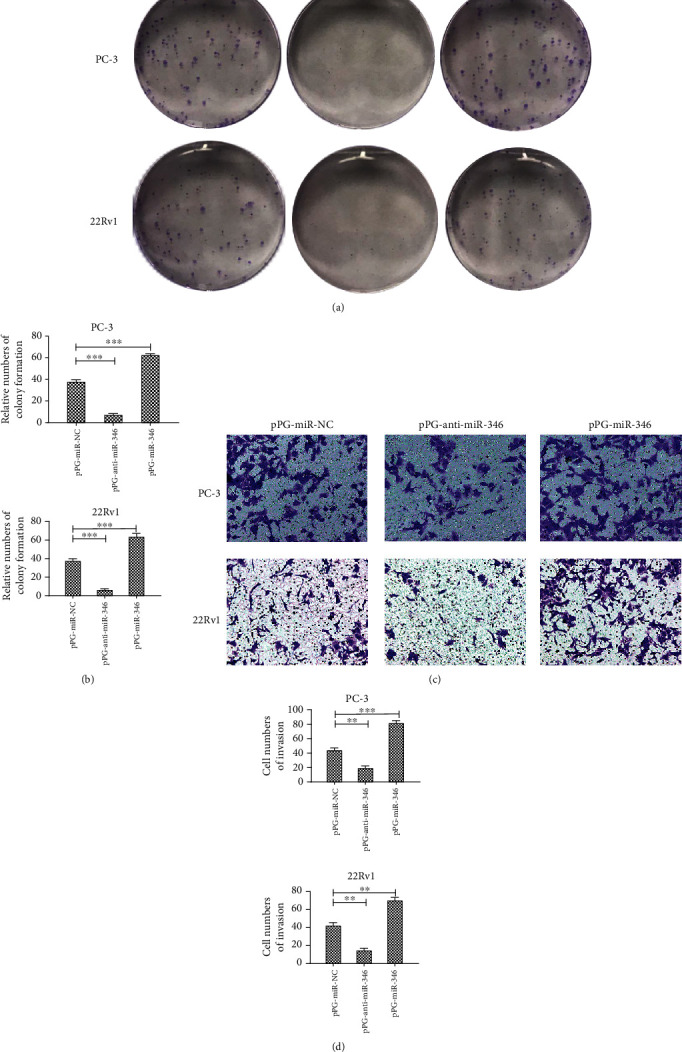
miR-346 enhances the proliferation and invasion of prostate cancer cells. (a) The colonizing ability of PC-3 and 22Rv1 cell lines transfected with pPG-miR-NC, pPG-anti-miR-346, or pPG-miR-346 was determined by colony formation assays. (b) Relative numbers of colony formation of PC-3 and 22Rv1 are shown. (c) The invasion of PC-3 and 22Rv1 cell lines transfected with pPG-miR-NC, pPG-anti-miR-346, or pPG-miR-346 was determined by transwell assays. (d) The cell numbers of invasion of PC-3 and 22Rv1 are shown. ^∗^*p* < 0.05, ^∗∗^*p* < 0.01, and ^∗∗∗^*p* < 0.001.

Recent studies reported that miR-346 promoted cell metastasis and EMT progression in other types of cancers [17–20]. However, the function of miR-346 in PCa heretofore had yet to be elucidated. In our study, we observed that the role of miR-346 in PCa cell proliferation and invasion was opposite to that of circDDX17 (Figure 6). Moreover, knockdown of miR-346 could reverse the effect of circDDX17 silencing on 22Rv1 and PC-3 cell lines (Figures 5(a) and 5(b)). These data revealed that miR-346 played an important role in PCa cell metastasis and EMT.

### 3.7. Knockdown of LHPP Promoted PCa Cell Metastasis and EMT Progression

**Figure 7:**
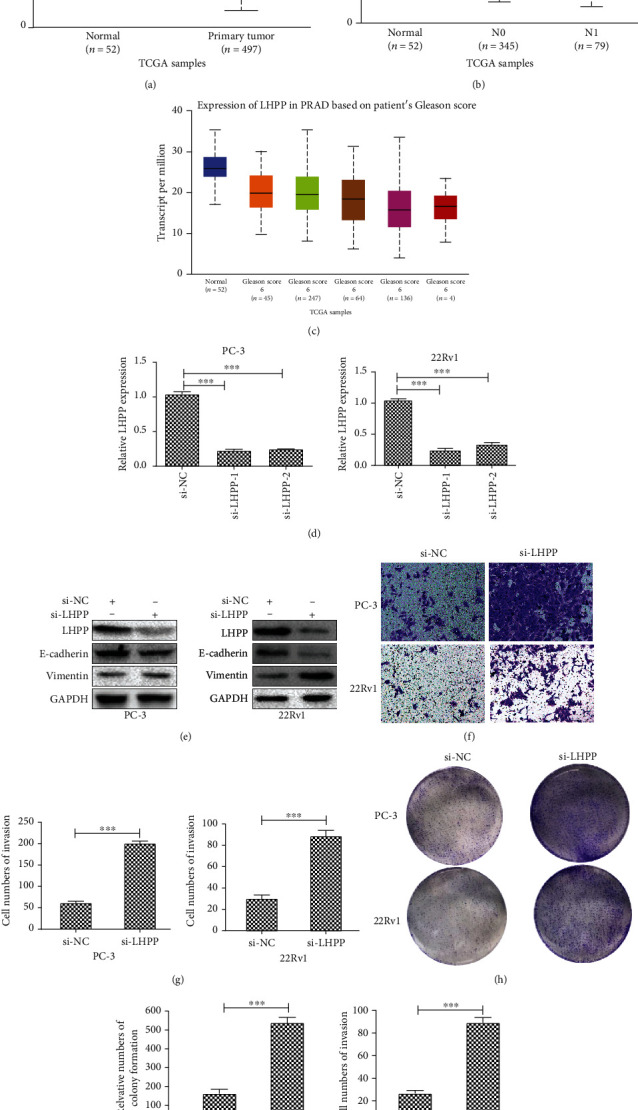
Silencing of LHPP promotes the EMT, invasion, and proliferation of prostate cancer cells. (a) LHPP expression profile in PCa and normal tissues was gained from TCGA database. *p* < 0.01. (b) LHPP expression profile in normal tissues, N0 PCa, and N1 PCa. N0-vs-N1: *p* < 0.01; normal vs. N1: *p* < 0.01. N0: no regional lymph node metastasis; N1: metastases in 1 to 3 axillary lymph nodes. (c) LHPP expression profile in normal tissues and Gleason score PCa. Gleason score 6 vs. Gleason score 9: *p* < 0.01; Gleason score 7 vs. Gleason score 9: *p* < 0.05; Gleason score 8 vs. Gleason score 9: *p* < 0.05. (d) Relative LHPP mRNA expression in PC-3 and 22Rv1 cell lines transfected with si-NC, si-LHPP-1, and si-LHPP-2. Si-LHPP-1 was used in follow-up experiments. (b) Relative LHPP, E-cadherin, and Vimentin protein expression in cell lines transfected with si-NC or si-LHPP was determined by Western blotting. (c) The invasion of 22Rv1 and PC-3 cell lines transfected with si-NC or si-LHPP was determined by transwell assays. (d) Relative cell numbers of invasion were calculated. (e) The colonizing ability of 22Rv1 and PC-3 transfected with si-NC or si-LHPP was determined by colony formation assays. ^∗^*p* < 0.05, ^∗∗^*p* < 0.01, and ^∗∗∗^*p* < 0.001.

Numerous studies have reported that LHPP can inhibit cell metastasis and EMT progression in various types of cancer cells [11, 12, 22]. Nevertheless, the role of LHPP in PCa cell metastasis and EMT progression had never before been illuminated. TCGA database was used to determine the levels of expression of LHPP in PCa-afflicted samples. LHPP expression was downregulated in PCa-afflicted compared to nonafflicted samples (*p* < 0.01) (Figure 7(a)). Interestingly, LHPP expression was downregulated in patients with an N1 grade of (metastases in 1 to 3 axillary lymph nodes) PCa compared to patients with an N0 grade of (no regional lymph node metastasis) PCa (Figure 7(b)). Furthermore, LHPP expression was reduced in the high Gleason score PCa treatment group (Figure 7(c)). These results revealed that LHPP expression was poorly predictive with respect to the stage of PCa.

To investigate the role of LHPP in PCa-afflicted cells, two LHPP-specific siRNAs were transfected into 22Rv1 and PC-3 cells. Figures 7(d) and 7(e) demonstrated that interference of LHPP mRNA and protein levels were observed in si-LHPP-1 and si-LHPP-2 cells. Thus, si-LHPP-1 was selected for use in further follow-up experiments. We performed Western blotting to evaluate EMT progression. An increase in mesenchymal marker Vimentin and a decrease in epithelial marker E-cadherin were each induced by transfection with si-LHPP in PCa-afflicted cells (Figure 7(e)). Transwell formation assays were used to assess the ability of invasion of PCa-afflicted cells. Results demonstrated that silencing of LHPP promoted the invasion ability of PCa-afflicted cells (Figures 7(f) and 7(h)). The colony formation assays confirmed that LHPP knockdown also promoted the colonizing ability of PCa-afflicted cells (Figures 7(h) and 7(i)). These data suggested that LHPP inhibited metastasis and EMT progression of PCa.

## 4. Discussion

circDDX17 was firstly reported as a tumor suppressor for colorectal cancer and in relation to silencing of circDDX17 such as to inhibit tumor metastasis [8]. Thus, we sought to assess and found that circDDX17 expression was significantly downregulated in PCa-afflicted sampled. We also found that circDDX17 had a negative effect on PCa cell metastasis and EMT. Therefore, we hypothesize that circDDX17 repressed metastasis and slowed the relative aggression of PCa by way of serving as an inducer of EMT. Moreover, we attempted to illuminate the underlying mechanism(s) by which manipulating circDDX17 expression occurred in PCa-afflicted cell lines.

Salmena et al. [23] was the first to have demonstrated a novel regulatory network across the transcriptome in which ceRNA activity serves as an important mediator in human cancers. The ceRNA network often acts as a reciprocal repression between lncRNAs and miRNAs. However, recent research has indicated that circRNAs are also involved in the ceRNA mechanism [24, 25]. Increasing lines of evidence also support that circRNAs may interact in a competitive manner with endogenous miRNAs by way of binding of their same sequence and thereby regulating the target genes of miRNAs. It has also been demonstrated that circFOXM1 can act as a sponge of miR-1304-5p such as to promote cell progression by regulating PPDPF and MACC1 in examinations of non-small-cell lung cancer-afflicted samples [26]. In another example, circLAMP1 was found to have facilitated T-cell lymphoblastic lymphoma progression by acting as a ceRNA such as to have targeted DDR2 by way of combining with miR-615-5p [27].The role of circDDX17 in PCa has however not heretofore been elucidated. Therefore, we investigated whether or not circDDX17 competed with miRNAs through the ceRNA mechanism. We found that overexpression of circDDX17 repressed miR-346 and that circDDX17 knockdown promoted miR-346, which subsequently was able to promote cell-colonizing abilities, ability of cellular invasion, and EMT progression in PCa-afflicted samples. Our study confirmed that miR-346 is upregulated in PCa-afflicted samples [20]. circDDX17 and miR-346 were expressed reciprocally in PCa-afflicted samples.

In contrast to the above findings, our study also revealed that miR-346 negatively regulated circDDX17. Thus, we hypothesized that miR-346 regulating the downregulation of circDDX17 was somewhat similar to the way in which miR-RNA-regulated silencing of protein-coding genes and that miRNAs posttranscriptionally inhibited gene expression by way of interacting with response elements of target genes. Ago, as a core component of RISC, is known to interact with miRNAs by which it regulates the expression of target genes [28]. To determine whether circDDX17 and miR-346 interacted in the same RISC that played an important role in RNA silencing, we performed RIP assays. Results indicated that circDDX17 was enriched in Ago2-containing beads. Furthermore, miRNA pull-down assays were performed, and we found that circDDX17 could be pulled down via biotin-labelled miR-346 in 22Rv1 cells. The dual-luciferase reporter assays also suggested that interactions between circDDX17 and miR-346 were functional. In accordance with the results above, we propose for the first time that circDDX17 could serve as a ceRNA for miR-346 in cases of PCa.

LHPP, an enzyme protein, is a recently discovered tumor suppressor. To our knowledge, we are the first to reveal that LHPP can repress PCa-afflicted cell metastasis and EMT progression both from a mechanistic and functional perspective. Notably, LHPP was downregulated in PCa-afflicted samples. We also observed that circDDX17 positively regulated the levels of endogenous LHPP mRNA and of expression of proteins by way of acting as a ceRNA for miR-346 in PC-3 and 22Rv1 cell lines.

circDDX17 was firstly reported to act as a tumor suppressor in examinations of colorectal cancer [8]. Thereafter, Ren et al. reported that circDDX17 reduced 5-fluorouracial resistance and hindered tumorigenesis in colorectal cancer by way of regulating miR-31-5p/KANK1 axis [29]. Likewise, circDDX17 was confirmed to play an important role in the dynamics underlying colorectal cancer [30]. In our efforts, we found that miR-346 was downstream of circDDX17. Thus, circDDX17 was able to act as a sponge for miR-346 such as to promote the levels of LHPP. Meanwhile, some targets of miR-346 have been reported in other studies and included NDRG2, YTHDF1, GSK-3*β*, and NFIB [31–34]. However, potential target(s) of circDDX17/miR-346 were not heretofore reported upon in the literature. In our own examinations, we found that LHPP was a target of circDDX17/miR-346.

In summary, we found that circDDX17 repressed metastasis and EMT progression in PCa-afflicted cells by way of acting as a sponge of miRNA, which consequently abolished the effect of miR-346. These effects acted to enhance the expression of LHPP. Thus, our findings suggested that the regulatory network of the circDDX17/miR-346/LHPP signaling pathway may be a potential prognostic and therapeutic target for improving diagnoses and treatments for PCa. The prognostic and the therapeutic value of circDDX17 in PCa should be verified in follow-up research.

## Figures and Tables

**Table 1:** Primer sets used for quantitative PCR.

Gene	Forward 5′-3′	Reverse 5′-3′
CircDDX17	TGCCAACCACAACATCCTCCA	CGCTCCCCAGGATTACCAAAT
miR-346	CACGGATCCCTTGTCAGCAAGGAGTG	CGGAATTCTAGGTTGGGAGCGAAGTG
U6	CTCGCTTCGGCAGCACA	AACGCTTCACGAATTTGCGT
GAPDH	AAGAAGGTGGTGAAGCAGGC	GTCAAAGGTGGAGGAGTGGG
LHPP	ACACGTCACTTGCCAGTCTCAC	CACAGGCTGTATGTCGCGGA

## Data Availability

Data and materials are available.

## Supplementary Materials

Supplementary Materials Figure S1: upregulation of circDDX17 suppresses the migration, EMT, and proliferation of prostatic cancer cells. (A) The circDDX17 expression in 22Rv1 cell lines transfected with pcDNA-NC or pcDNA-circDDX17 was determined by qPCR. (B) Relative cell proliferation in 22Rv1 cell lines transfected with pcDNA-NC or pcDNA-circDDX17 was detected by CCK8. (C) The invasion of 22Rv1 cell lines transfected with pcDNA-NC or pcDNA-circDDX17 was determined by transwell assays. (D) Relative cell numbers of invasion in 22Rv1 cell lines were shown. (E) The colonizing ability of 22Rv1 cell lines transfected with pcDNA-NC or pcDNAcircDDX17 was determined by colony formation assays. (F) Relative cell numbers of colony formation in 22Rv1 cell lines were shown. (G) Relative quantification of Vimentin IF images. (H) Relative quantification of E-cadherin IF images. ^∗^*p* < 0.05, ^∗∗^*p* < 0.01, and ^∗∗∗^*p* < 0.001.
