# Supplementary figures and images for: The Significance of Circular RNA DDX17 in Prostate Cancer
Source: Biomed Res Int. 2020 Aug 20;2020:1878431. doi: 10.1155/2020/1878431 (PMC7456467; doi:10.1155/2020/1878431)

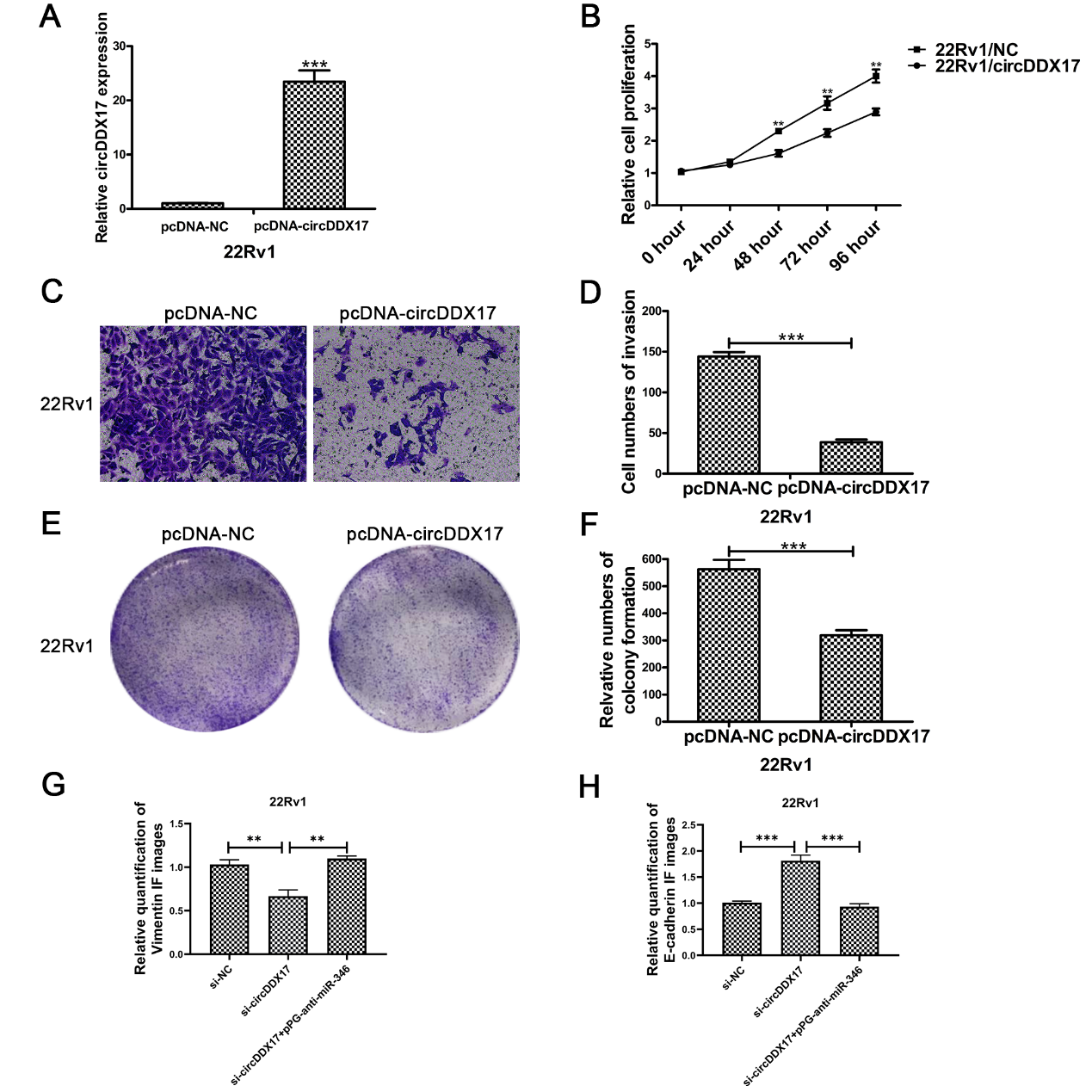

Supplement: Supplementary Materials — Figure S1: upregulation of circDDX17 suppresses the migration, EMT, and proliferation of prostatic cancer cells. (A) The circDDX17 expression in 22Rv1 cell lines transfected with pcDNA-NC or pcDNA-circDDX17 was determined by qPCR. (B) Relative cell proliferation in 22Rv1 cell lines transfected with pcDNA-NC or pcDNA-circDDX17 was detected by CCK8. (C) The invasion of 22Rv1 cell lines transfected with pcDNA-NC or pcDNA-circDDX17 was determined by transwell assays. (D) Relative cell numbers of invasion in 22Rv1 cell lines were shown. (E) The colonizing ability of 22Rv1 cell lines transfected with pcDNA-NC or pcDNAcircDDX17 was determined by colony formation assays. (F) Relative cell numbers of colony formation in 22Rv1 cell lines were shown. (G) Relative quantification of Vimentin IF images. (H) Relative quantification of E-cadherin IF images. ∗p < 0.05, ∗∗p < 0.01, and ∗∗∗p < 0.001. [file 1878431.f1.docx]
